# A New Concept on Chemotherapy-Induced Nausea and Vomiting: Persian Medicine Viewpoint

**DOI:** 10.31661/gmj.v8i0.1412

**Published:** 2019-05-09

**Authors:** Ali Aminian, Seyde Sedighe Yousefi

**Affiliations:** ^1^Department of Traditional Persian medicine, Faculty of Medicine, Mazandaran University of Medical Sciences, Sari, Iran

**Keywords:** Persian Medicine, Nausea, Vomiting, Chemotherapy, Side Effect

## Dear Editor,

Unfortunately, chemotherapy-induced nausea and vomiting (CINV), is one of the most common and bothering side effects, which plays a significant negative role in participants’ cooperation and even quality of life [1]. CINV has a complex mechanism results from the physiological collaboration and interaction between the gastrointestinal (GI) tract and central nervous system (CNS), by the splits of the afferent and efferent vagus nerve [2]. In Persian medicine (PM), nausea is commonly known as “*Ghathayan*” that means the GI attempt to expel the substances accumulated in the stomach, without success, and vomiting is known as “*Ghey*” means GI motility in order to repel the accumulated substances through the mouth successfully [3]. Both of these definitions are corresponding to the states of nausea and vomiting in modern medicine [4]. According to the holistic viewpoint of PM, the human body is made up of four main substances called “Humor” (*Khelt*), each of which has a unique temperament (*Mizaj*), and, on the basis of temperament, they play certain roles in the body. Normal humors naturally produce in the liver, and those produce outside, including the stomach, are almost abnormal and known as a type of disease named dystemperament or “*su-e-mizaj*.” Between humors, yellow bile(*Safra*) has “hot and dry” temperament, which in abnormal type, causes the most signs, symptoms and also severe complications among the organs, such as the stomach. PM philosophies emphasized on the plurality of nausea and vomiting in individuals with hot and dry or choleric temperament (*Mizaj-e-safravi*) [5]. According to new findings, the risk of CINV is higher in individuals with genetic variation of being rapid metabolizer, which, in PM means a person who owns hot and Choleric emperament (*Safravi*) [6]. It means, abnormal yellow bile, which is produced or accumulated in the stomach, acts as a pathogenic factor, causes inflammation and irritation in the mucosal layer of the stomach, especially the cardia where is full of nerve fibers [7]. Comparison of the structural and functional properties between abnormal yellow bile and free radicals, which produce by chemotherapy agents and cause GI mucosal layer damage, reveals similarities as shown in Table-1. It seems that free radicals are considered to be a kind of abnormal yellow bile in PM [4]. On the other hand, In PM viewpoint, important organs have close physiological links and interactions with each other, so that the health and illness of each one can directly affect another. This feature is known as the “organ contribution” (*Mosharekat*) law [9]. One of the most important organ contributions is the participation of the stomach and brain, which is equivalent to the functional and physiological pathway, known as the Gut-Brain axisin Modern medicine [4]. In recent studies, the role of this axis has been proven, in the prevalence of CINV, in patients with ovarian cancer [6]. So, we can describe that chemotherapy agents, especially cytotoxic drugs, produce free radicals in the GI tract, especially in the stomach and upper intestine, which have similar characteristics and functions to abnormal yellow bile. By producing free radicals, damage occurs in the DNA of the entrochromaphenic cells, which are located and spread in the stomach. Entrochromaphenic cells contain a variety of neurotransmitters, including serotonin. By damage of entrochromaphenic cells, these neurotransmitters excrete and stimulate the adjacent terminal receptors of the abdominal vagus nerve, such as the serotonin receptor, so the messages send through the branches of the abdominal vagus nerve to the nausea center in brain stem, then to chemoreceptor trigger zone, and after the final analysis, the feeling of nausea, vomiting perceives by the cerebral cortex [10]. This bi-directional and physiological route is the same participatory route as organ contribution law. It seems that the joint operation of the GI tract, including post-chemotherapy production and accumulation of free radicals in the stomach, with similar characteristics to abnormal yellow bile, in one hand, and performance of the physiological pathway named Gut-Brain axis, which is similar to the organ contribution law in PM, on the other hand, explains CINV physiopathology in PM viewpoint. Consequently, due to this functional adaptation, it seems to be beneficial to search more about the recommendations of the PM scholars about nausea and vomiting induced by abnormal yellow bile, for relative improvement in cancerous patients, whom often not well-protected by current anti-emetic drugs, alone.

## Acknowledgment

This article was driven from a Ph.D. thesis sponsored by Mazandaran University of Medical Sciences, Sari, Iran. IRCT Code: IRCT20171118037516N1

## Conflict of Interests

The authors declare that there is no conflict of interest.

**Table 1:** Comparison Between Yellow-Bile and Free Radicals Specifications

** Free Radical [8] **	**Similarities**	** Yellow Bile [3, 5, 9] **
Rapid, Intensive response to biological targets	*	Subtle, High heat, Fast penetration
Molecular-sized objects	*	Intensity and velocity of breaking down to the smallest components due to heat
Very short lifespan	*	Quick dissolving
